# Imaging biological macromolecules in thick specimens: The role of inelastic scattering in cryoEM

**DOI:** 10.1016/j.ultramic.2022.113510

**Published:** 2022-07

**Authors:** Joshua L. Dickerson, Peng-Han Lu, Dilyan Hristov, Rafal E. Dunin-Borkowski, Christopher J. Russo

**Affiliations:** a MRC Laboratory of Molecular Biology, Francis Crick Avenue, Cambridge CB2 0QH, UK; b Ernst Ruska-Centrum für Mikroskopie und Spektroskopie mit Elektronen, Forschungszentrum Jülich GmbH, 52425 Jülich, Germany

**Keywords:** TEM, Low dose imaging, *C*_*c*_ correction, cryoEM, Phase contrast, Specimen induced decoherence

## Abstract

We investigate potential improvements in using electron cryomicroscopy to image thick specimens with high-resolution phase contrast imaging. In particular, using model experiments, electron scattering theory, Monte Carlo and multislice simulations, we determine the potential for improving electron cryomicrographs of proteins within a cell using chromatic aberration ($C_{c}$) correction. We show that inelastically scattered electrons lose a quantifiable amount of spatial coherence as they transit the specimen, yet can be used to enhance the signal from thick biological specimens (in the 1000 to 5000 Å range) provided they are imaged close to focus with an achromatic lens. This loss of information quantified here, which we call “specimen induced decoherence”, is a fundamental limit on imaging biological molecules *in situ*. We further show that with foreseeable advances in transmission electron microscope technology, it should be possible to directly locate and uniquely identify sub-100 kDa proteins without the need for labels, in a vitrified specimen taken from a cell.

## Highlights

• Potential improvements in cryoEM of thick specimens are investigated.
• The theory of specimen induced decoherence is developed in the context of biological cryoEM.
• The use of *C*_*c*_ correction, liquid helium cooling and phase plates for imaging thick biological specimens is considered.
• Conclusions about the minimum size molecule potentially identifiable in situ without labels are drawn.

## 1 Introduction

Single-particle electron cryomicroscopy (cryoEM) can be used to determine the atomic structure of biological molecules and macromolecular assemblies whose masses range from a few tens of thousands to millions of Daltons [1]. High-resolution imaging requires that the specimen is embedded in a layer of amorphous water ice which is as thin as possible, ideally just thicker than the diameter of the molecule or complex itself. Structure determination by cryoEM usually begins with biochemical isolation, purification, and concentration of the molecules of interest, thus creating a specimen appropriate for vitrification in a monolayer by the Dubochet cryoplunging technique [2]. The vast majority of these specimens are thus 100 to 400 Å thick, commensurate with their mass in the ten kiloDalton to megaDalton range.

As structural biology accumulates more and more atomic structures [3], and the ability to use previous structures to predict unknown structures related by evolution or denovo improves [4], an increasingly important frontier of electron cryomicroscopy is the imaging of biological molecules within their native environments. In this context the goal can be to determine a structure by a process called sub-tomogram averaging, which can be considered a modified form of single particle cryoEM that incorporates varying amounts of data from tilted specimens into the 3D reconstruction process [5]. This is particularly useful for targets not amenable to purification and isolation, but the fundamental problems associated with imaging a tilted specimen mean this will remain more difficult in most cases than determining the structures from purified specimens by single particle cryoEM. This was made particularly clear from recent work on the SARS-CoV-2 virus [6], where both cryoEM and electron cryotomography (cryoET) were used to determine the structure of the spike protein with great speed. Increasingly, the goal of cryomicroscopy of cellular specimens will likely shift to identifying the particular molecules and structures present in a cryogenically preserved portion of a cell or organelle. This will include direct identification of the position and orientation of a macromolecule relative to other structures in the cell and potentially even definitive identification of the specific conformation it is in. This has long been the goal of cryoET, in which a series of tilted cryomicrographs are collected and reconstructed into a 3D tomogram [7], [8], and has already been realised for ribosomes [9], [10], [11]. Recently, the need for tilting the specimen at all has been called into question as the position, orientation and depth of molecules in a cellular specimen whose structure is known can be found by cross correlation with reference projections in a single 2D cryomicrograph [12].

**Fig. 1:**
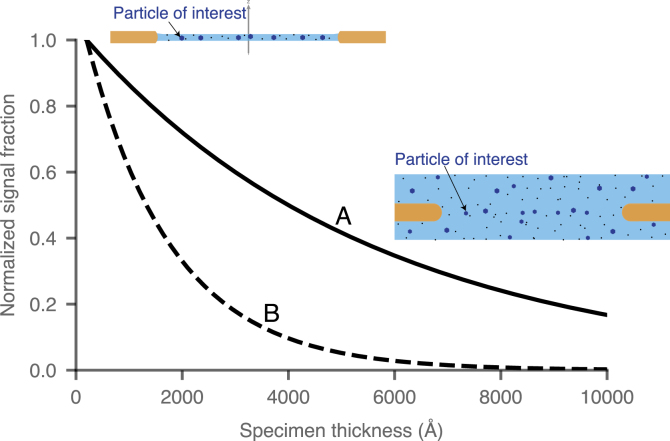
Comparison of simulated signal from a particle of interest as a function of specimen thickness for 300 mg/ml of protein in amorphous ice. The accelerating voltage is 300 kV. **A** is the fraction of electrons that have undergone a single elastic scattering event and **B** are those that have undergone a single elastic scattering event and not been inelastically scattered. The elastic scattering cross sections were taken from the NIST electron elastic-scattering cross-section database [16] and the inelastic scattering cross sections calculated from electron energy loss spectroscopy (EELS) measurements [17]. The electron fractions were calculated using Poisson statistics. The y-axis is normalised to be 1.0 for protein embedded in 300 Å thick amorphous ice.

The aim of visualising macromolecules in their cellular context necessitates that vitrified cellular specimens prepared for either cryoET or 2D template matching are thicker than those prepared for single-particle cryoEM. This is a major limitation on the obtainable signal and resolution (Fig. 1) since it results in a larger proportion of electrons lost to inelastic scattering, and to a lesser extent, multiple elastic scattering [13]. Electrons that inelastically scatter have, by definition, lost significant energy, and are thus incorrectly focused in the image plane due to the chromatic aberration ($C_{c}$) of the objective lens. As a result, inelastic electrons that carry elastically scattered information in their wavefront do not provide high resolution information in phase contrast images and will instead contribute noise. The current practice in both single particle cryoEM and cryoET is to remove these electrons with an electron energy filter to reduce that noise. Given the advent of practical chromatic aberration correction [14], there is a potential improvement in signal by incorporating these inelastically scattered electrons in the image, yet the details of how much improvement is possible are unknown. Quantifying the potential improvement and requirements for imaging thick specimens using both elastic and inelastic electron scattering for phase contrast by cryoEM is our focus in this work. In an accompanying paper, we address another related question that has been controversial in the literature: whether the information available from phase contrast images of an individual particle embedded in a thicker specimen depends on its position relative to the electron beam entrance vs. exit plane [15]. We experimentally find that the ability to resolve a particle by phase contrast does not depend on its depth within the specimen and thus take that as a given in the theory presented below.

## 2 Theoretical background

Inelastic scattering changes both the energy and direction of electrons; the latter can be thought of as reducing their spatial coherence. High resolution imaging of proteins relies on phase contrast generated by the superposition of electron waves in the image plane. At low scattering angles, and for weakly scattering materials like carbon and water, phase contrast is typically generated using the applied defocus and the spherical aberration of the objective lens. The phase shift, W, is given by the wave aberration equation

(1) $$W\left(\theta \right)=\frac{\pi }{2\lambda }\left(2\Delta z\theta^{2}+C_{s}\theta^{4}\right)$$

where θ is the angle the electron trajectory makes to the optical axis, λ is the electron wavelength, Δz is the defocus (with underfocus being negative), and $C_{s}$ is the spherical aberration coefficient [18]. The spatial coherence of the electron source is determined by the effective source size, whose effect on the signal is characterised by the spatial coherence envelope function [19], [20]

(2) $$E_{s}\left(q\right)=\exp\left[-{\left(\frac{\pi \alpha }{\lambda }\right)}^{2}{\left(\lambda \Delta zq+C_{s}\lambda^{3}q^{3}\right)}^{2}\right]$$

where $E_{s}\left(q\right)$ is the fractional amplitude at frequency q, and α is the semi-angle of the source electron distribution, defined as the value where it reduces to 1/e of its value at the origin.

Inelastic scattering will have the effect of increasing the angular distribution of the electrons, hence increasing the effective source size and reducing the fractional amplitude at non-zero frequency. Ferrel used the Bohm–Pines electron plasma theory to derive the angular distribution of collective excitations [21], which are the source of most inelastic scattering in materials like amorphous water and carbon [22]. The angular distribution can be approximately described by the differential inelastic scattering cross section

(3) $$\frac{d\sigma_{in}}{d\Omega }\propto \frac{1}{\theta_{E}^{2}+\theta {}^{2}}\;\;\;\;\text{with}\;\;\theta {}_{E}=\frac{\Delta p}{p}$$

where θ is the angular deflection, Δp is the loss of momentum of the inelastically scattered electron, and p is the magnitude of the initial momentum [21].

Several studies have used inelastic electron holography to investigate the degree of coherence of inelastically scattered electrons [23], [24], [25], [26]. These studies confirmed that coherent phase contrast is observable within an inelastically scattered wave. Furthermore, these experiments showed that the spatial coherence, and thus the phase contrast, decreased as the energy loss increased, which is also consistent with the momentum term in Eq. (3). To make accurate predictions of the phase contrast achievable from inelastically scattered electrons generated in thick biological specimens, experimental measurements of the contrast loss in relevant specimens as a function of defocus are necessary.

With this background in mind, we next describe experiments to measure the angular distribution of inelastically scattered electrons generated upon transit through model amorphous carbon specimens of known thickness, as this is the key unknown parameter in theoretically describing the loss of information from thick specimens. These include imaging gold particles on amorphous carbon specimens of varying thickness, under different imaging conditions, with varying amounts of energy loss, and the use of a chromatic aberration corrected microscope (PICO in Jülich [27]). We then use these measurements to determine, by theory and simulation, the extent to which the resultant spread of angles results in a loss of spatial coherence, and how much improvement in signal is possible with the use of $C_{c}$ correction and energy filtered imaging. Note that the spatial coherence of a typical Schottky emitter with brightness of order $10^{7}$ A/m${}^{2}$/sr/V, under the conditions used for low-dose imaging of cryogenically preserved specimens (∼ 2 e${}^{-}$/Å${}^{2}$/s), has an illumination semiangle of less than 1 μrad, and can be used at several micrometres of defocus without loss [28]. With these measurements in hand, we then evaluate the conditions necessary to best improve the imaging of thick biological specimens using inelastically scattered electrons, and what this potential improvement implies for future technological developments.

## 3 Materials and methods

### 3.1 Specimen preparation

Model specimens were created consisting of 100 Å diameter gold particles dispersed onto foils of amorphous carbon of several different thicknesses on TEM grids. We chose this specimen since carbon has a similar mass thickness to that of amorphous ice, but it is radiation resistant and allows us to perform experiments at room temperature. Amorphous carbon foils of several different thicknesses were created using vacuum deposition from heated carbon rods onto freshly cleaved 75 × 25 mm mica sheets (Agar Scientific) in a high vacuum chamber (Edwards 306A). A sharpened rod was pressed against another flat rod with a spring and the junction was positioned 120 mm from the mica. The system was pumped to a base pressure of $5\times 10^{-5}$ mbar and current was applied across the rod assembly for varying amounts of time to deposit different thickness carbon foils. After deposition, the density of the resulting amorphous carbon films was measured by floating the carbon onto a glass slide and scraping flakes of it off into a bromoform/chloroform density gradient column. The column provides a smooth density gradient from bromoform at the bottom (2.89 g/cm^3^) to chloroform at the top (1.49 g/cm^3^). The flakes are left to settle in the column and the density of liquid at their final position corresponds to the density of the flakes, measured at 1.7 g/cm^3^ in this instance. The thickness of the amorphous carbon was measured by cleaving an area from mica using adhesive tape (Scotch® Crystal). The difference in height across the step edge of the cleaved area was measured using an atomic force microscope (Asylum Research MFP3D) in direct contact mode. The process was repeated using carbon that had been floated onto a 6 mm diameter sapphire disk (Wohlwend Art.616) to ensure consistent results. Suspended foils were then prepared with the characterised carbon by transferring the carbon onto the flat side of 300 line per inch square mesh gold grids (Agar Scientific) by flotation on water [29]. After transfer, the grids were gently heated on a hot plate until there was visible flattening of the foil, which occurred after around 10 min at 200°C, to improve the contact between the carbon and the grid. The grids were then exposed to a low energy plasma (Fischione 1070) comprising a mixture of argon and oxygen (19:1) for 15 s at 70% power. The source gases were N6.0 grade (BOC) and the plasma treatment was used to render the surface of the carbon hydrophilic and remove surface contaminants. Based on the previously calibrated etch rate for these conditions [29], the plasma treatment etched less than 10 Å from each surface of the carbon. A 50μl solution of 100 Å gold particles (BBI) at an optical density of 100 was dispersed by sonicating for a few seconds using a probe ultrasonicator (Kontes KT50 micro ultrasonic cell disruptor, frequency 20 kHz) at an output amplitude of 60%. Three microlitres of solution were then immediately pipetted onto the carbon side of each grid. The grids were blotted with filter paper (Whatman No. 1) to remove excess liquid and were then left to dry in air before being stored in glass petri dishes until they were transferred to the electron microscope. For the $C_{c}$ corrected experiments, this included transport to Germany in a grid storage box.

### 3.2 Electron microscopy

The power in the reflection from the 111 lattice planes of the gold particles (at 2.35 Å) was measured as a function of defocus according to the method described in Ref. [30]. To increase throughput, a custom script was written in SerialEM [31] to change the defocus, position, and set the conditions of the energy filter. Data was collected at defocus steps of 250 Å on a transmission electron microscope operating at 300 kV (FEI Titan Krios G2 with X-FEG). The spectrometer setup comprised a Gatan Quantum energy spectrometer with a Gatan K2 direct electron detector. The width of the energy selection slit was set to 6 eV and was centred on the zero energy loss peak (ZLP), or at the plasmon peak (23 eV) or at other energy loss values as detailed in the results. Data was collected at a nominal magnification of 165,000×, corresponding to a magnified pixel size of 0.66 Å at the specimen. Fluxes ranged from 3–10 electrons/px/s, with an exposure time of 4 s and binning 1 on the detector. Data was collected for specimens with 200 Å and 2000 Å thick carbon with the gold particles on top of the carbon (on the surface closer to the electron source).

Electron energy loss spectroscopy (EELS) data was collected using a transmission electron microscope operated at 300 kV (FEI Polara G2), and an electron spectrometer (Gatan Tridiem 864) with a 4k × 4k phosphor-coupled CCD to record the spectra (Gatan US4000). The energy dispersion was calibrated using an evaporated aluminium film (Electron Microscopy Sciences), which has a sharp plasmon peak at 15.1 eV at room temperature. Spectra were acquired in imaging mode without an objective aperture to ensure a high collection angle. The total fluence was kept low to prevent saturation of the zero-loss peak.

Images were also collected on a $C_{c}$ corrected microscope, the FEI Titan 50–300 PICO [27], operating at 200 kV for the 100 Å gold particles on 2000 Å thick carbon (Fig. 6).

**Table 1:** The average full width at half maximum (FWHM) as a function of defocus of the power in the gold 111 reflection by experiment and simulation. The fading of the power with defocus was measured experimentally for inelastically scattered electrons with different energy losses. N is the number of particles used at each experimental energy and the error reported is the 95% confidence interval (see Fig. 4 for examples). The experimental values are compared to those determined with simulations using different methods (Sections 3.5.1, 3.5.2, 3.5.3)

	Experimental		Simulated FWHM		
Energy loss	FWHM	N	CTF	Envelope function	Multislice
9 eV	5600 ± 800 Å	10	5200 Å	5600 Å	5800 Å
15	3400 ± 500	9	3400	3700	3500
23	2600 ± 200	13	2400	2600	2200
30	1800 ± 300	11	2000	1600	1700

### 3.3 Data analysis and processing

Image stacks were aligned using Unblur [32] to remove thermal drift and the defocus was estimated using CTFFIND [33]. The particle and both sidebands were boxed out [34] and the intensity of the 2.35 Å resolution reflection was measured from the Fourier transform [30]. For the EELS data, the background was removed from the pixels by subtracting the average pixel value on the detector just above the spectrum. The spectra measured with no specimen present in the beam were averaged and then superimposed over each energy loss spectrum. The integrated counts outside of the source spectrum were then taken as the number of inelastically scattered electrons. A spectrum from the thinnest specimen (200 Å thick carbon) was measured to provide a single inelastic scattering energy loss spectrum for subsequent simulations, and allowed us to roughly measure the mean free path length in the carbon films as prepared. Given a measured path length of 2400 Å, 96% of the energy loss electrons will be from a single inelastic scattering event and so this is a reasonable approximation. The energy loss spectrum from the 200 Å thick carbon was also used to simulate energy loss spectra from amorphous ice and protein, assuming single inelastic scattering events in these materials have approximately the same energy loss spectrum as the carbon. At a beam energy of 300 keV, an inelastic mean free path length of 3140 Å was taken for amorphous ice [17] at a density of 0.93 g/cm^3^ [35]. The inelastic scattering cross sections for atoms in a protein were scaled from elastic scattering cross sections according to the experimental ratio determined in Ref. [36].

### 3.4 Envelope function

The spatial coherence envelope function for a Gaussian angular spread was derived by Frank in Ref. [19] and is reproduced in Eq. (2). Using the same method, this formula can be re-derived for the Lorentzian distribution as an improved approximation for inelastically scattered electrons. This derivation is shown in Appendix A, and gives the following formula for the spatial coherence envelope

(4) $$E\left(\vec{k}\right)=\int_{0}^{q_{c}}\frac{\gamma }{{\left(q^{2}+\gamma^{2}\right)}^{\frac{3}{2}}}J_{0}\left(2\pi q\left(\Delta z\theta +C_{s}\theta^{3}\right)\right)q\;dq$$

where q is the spatial frequency, γ is the half width at half maximum, $J_{0}$ is the 0th order Bessel function, and $q_{c}$ is the cutoff for plasmon scattering, which is estimated to be $\sqrt{2\theta_{E}}$ [37]. For the results below, integration was performed numerically using the experimentally determined parameters.

### 3.5 Simulations

To begin, the fading of the 2.35 Å reflection from gold 111 as a function of defocus for a particular electron energy loss was simulated and compared with the experiments described above. Having verified the approach was sound, the simulations were then extended to a range of resolutions important for biological imaging. This used a continuum approximation for 300 mg/mL protein embedded in amorphous ice where the density of the mixture was taken as 1.06 g/mL. This then allowed separation of the specific effects of increasing defocus on a $C_{c}$ corrected image without energy filtering. Three methods of simulation were used, and are detailed in turn below.

#### 3.5.1 Contrast transfer function (CTF) simulations

Monte Carlo simulations of electrons transiting specimens of varying thickness were performed in the following way:

1. For the given specimen thickness, a Monte Carlo simulation is performed to produce an EEL spectrum. After every inelastic scattering event, an energy loss is sampled from the thin (200 Å) EEL spectrum. Upon completion of transit through the sample, the energy loss as well as the number of inelastic scattering events of each electron is recorded.
2. For each of $10^{7}$ simulated electrons, a source angle was chosen from a Gaussian distribution with a FWHM of 0.01 mrad, which was determined by fitting Eq. (2) to the data for zero loss electrons. This is slightly higher than in Ref. [30] as a result of the higher fluxes used for this study.
3. An energy loss for each electron was chosen from experimental EELS data by inverse transform sampling. If spectra for a particular thickness had not been experimentally measured, a simulated energy loss spectrum was used.
4. If energy filtering was applied, a particular electron was only counted if its energy loss fell within the energy range of the slit.
5. The probability that an energy loss arose from n inelastic scattering events, where n is between 0 and 6, was calculated from the simulated EEL spectrum produced in step 1 by inverse transform sampling.
6. For each inelastic scattering event, the angle of scattering was also sampled using Eq. (3). A limit was placed at the maximum scattering angle for a plasmon excitation of $\sqrt{2\theta_{E}}$ which allowed sampling from a bounded Lorentzian function.
7. The phase shift for each electron at a peak around 2.35 Å resolution was calculated from Eq. (1) at every defocus value between +10000 Å and −50000 Å in 100 Å steps.
8. The CTF was taken as the sine of the phase shift and the average absolute CTF over all electrons was calculated for each defocus value.

#### 3.5.2 Numerical simulations

The numerical simulations were performed in the same way as in the previous section. For these simulations, instead of calculating the phase shift for a given energy loss electron in step 7, the envelope function was calculated by numerical integration from Eq. (4). The signal fraction was then taken as the average envelope over all electrons.

#### 3.5.3 Multislice simulations

The multislice simulations were performed in the following steps:

1. Electron wavefunction simulations were performed using temsim [38] to produce an exit wavefunction from 100 Å gold particles.
2. A CTF with varying amounts of defocus, between +10000 Å and −50000 Å in 100 Å steps, was applied to the exit wavefunction to produce an image.
3. Inelastic scattering was simulated by applying angular shifts of varying amounts to the exit wavefunction prior to imaging.
4. These images were then combined via a weighted average according to the Lorentzian distribution of inelastic scattering (Eq. (3)).
5. The intensity of the 2.35 Å resolution reflection was then calculated from the Fourier transform of each combined image.

## 4 Results

**Fig. 2:**
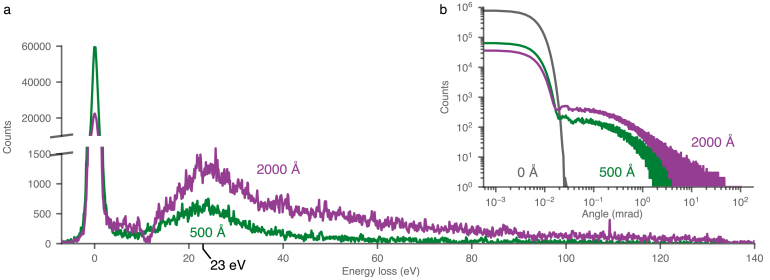
Inelastic scattering of 300 keV electrons through amorphous carbon. The electron energy loss spectra (a) from amorphous carbon of thicknesses 500 Å and 2000 Å show a most probable loss energy of 23 eV. 56% and 19% of the primary 300 keV electrons lose between 6 and 140 eV during transit through the 2000 Å and 500 Å thick foils respectively. Inset (b) shows the angular distribution of all electrons imaged with a Schottky FEG for carbon of thicknesses 500 Å and 2000 Å (note the log–log scale). The angular spread is calculated using the experimental EEL data in (a) and Eq. (3).

As electrons transit a biological specimen, they suffer scattering events that result in a loss of energy and a change in direction. These events increase in frequency as the specimen becomes thicker. The energy loss spectra for thin (500 Å) and thick (2000 Å) amorphous carbon specimens were measured using EELS (Fig. 2a). The most probable loss was found to be 23 eV, with a 3 fold increase in the number of inelastically scattered electrons for a 4 fold increase in thickness. From these energy distributions, the angular spread of the beam post transit was determined using Eq. (3) and a Monte Carlo simulation (Fig. 2b). These show that there is a drastic increase in the angular spread of the inelastic electrons even when transiting a moderately thick specimen where multiple scattering can still be neglected (less than 2 mean free path lengths thick). Furthermore, these energy loss distributions can be used to guide Monte Carlo simulations of a range of thicknesses.

**Fig. 3:**
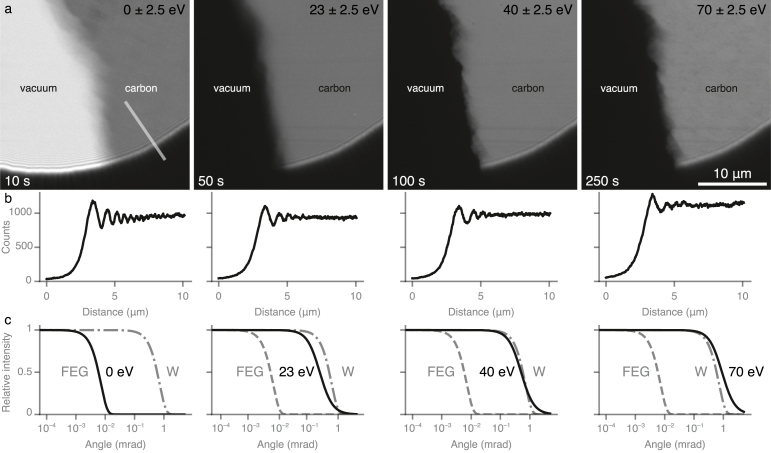
Fresnel interference fringes produced in energy filtered images by the edge of a 70μm condenser 2 aperture positioned over a 2000 Å thick amorphous carbon foil near a torn edge. Electrons of different energy losses are selected using an energy filter with a 5 eV slit width. Each column corresponds to a particular energy loss value (0 eV, 23 eV, 40 eV & 70 eV); the electron flux on the specimen is the same in each image, with the exposure times noted. Row (b) shows a section on the carbon region and across the edge of the aperture for each energy filtered image. Note the loss of phase contrast fringes as the energy loss increases. Row (c) shows the corresponding simulated angular distribution for each energy loss window, and is plotted with the angular spread of the Schottky field emission gun under the conditions used for the experiment and a tungsten hairpin source for comparison.

The increased angular spread caused by transit through specimens of this thickness range is bound to have an appreciable effect on the spatial coherence, and thus the signal, in phase contrast micrographs. This effect is easily demonstrated with a simple experiment shown in Fig. 3, where the phase interference fringes from the edge of a condenser aperture are imaged over a 2000 Å thick carbon foil at different energy loss values. As the energy loss increases, the magnitude of the Fresnel fringes drops (Fig. 3b). In effect, the thick specimen reduces the spatial coherence of the inelastically scattered electrons by an amount roughly equivalent to switching from a Schottky field emitter to a tungsten source (Fig. 3c). This effect is independent of the optics of the microscope and is therefore unavoidable.

**Fig. 4:**
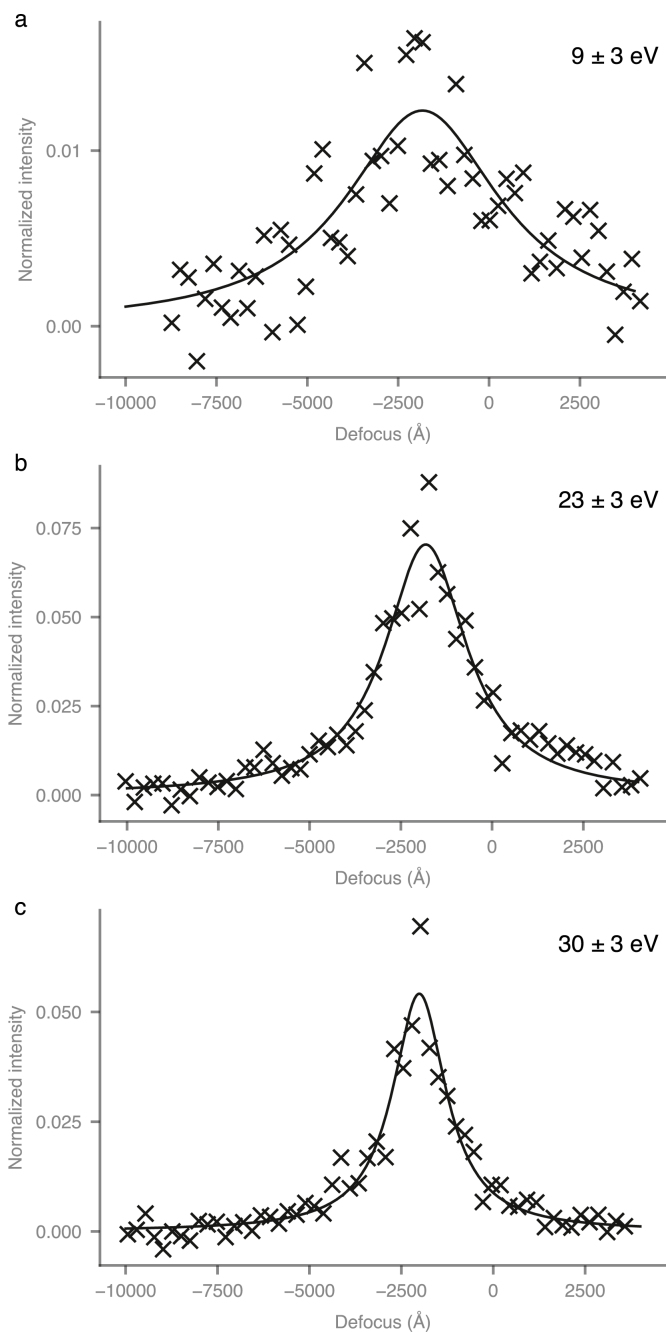
Measured power in the 2.35 Å gold 111 reflection vs. defocus for different energy loss electrons. Points are from a single representative measurement collected from a 100 Å diameter gold particle on a 2000 Å thick carbon film. The energy selection slit was centred on 9, 23 & 30 eV for (a), (b) & (c) respectively. Curves are a Lorentzian fit to the data. The y-axis is normalised to the corresponding fraction of electrons in the EEL spectra (Fig. 2).

To quantify this loss, we turned to a method of measuring spatial coherence developed previously in the context of studying charging induced decoherence [30]. The power in the gold 111 reflection from a particle is measured as a function of defocus; when plotted, this falloff then quantifies the spatial coherence. We extended this method to measure the spatial coherence of inelastically scattered electrons by measuring the intensity of the reflection as a function of defocus in a specific energy loss window. Defocus series were collected from strongly diffracting gold particles on a 2000 Å thick carbon foil using an energy selection slit of 6 eV in width, centred on particular energy loss values (Fig. 4 and Table 1). In particular, the fading of reflections from many particles at 9, 15, 23 and 30 eV energy loss was quantified; representatives are shown in Fig. 4a–c. The maximum intensity in each dataset was at a defocus where the first derivative of the wave aberration function (Eq. (1)) is zero, which occurs at a defocus of −1900 Å for 300 keV electrons and a $C_{s}$ of 2.7 mm. The fading of the intensity in each dataset was fitted to a Lorentzian, and these were then used to calculate the mean and 95% confidence intervals in the statistics reported in Table 1.

The experimental values were then compared to those from three types of simulations: 1. Monte Carlo simulations using the CTF as detailed in Section 3.5.1, 2. Monte Carlo simulations using numerical envelope functions (Section 3.5.2) and 3. Multislice simulations of images (Section 3.5.3). All methods of simulating the loss of contrast corroborate well with the experimental values (see Table 1); the simulations using envelope functions showed the best agreement by a small margin, and were the easiest of the three to calculate so were used for subsequent analysis of a range of different potential biological specimens.

**Fig. 5:**
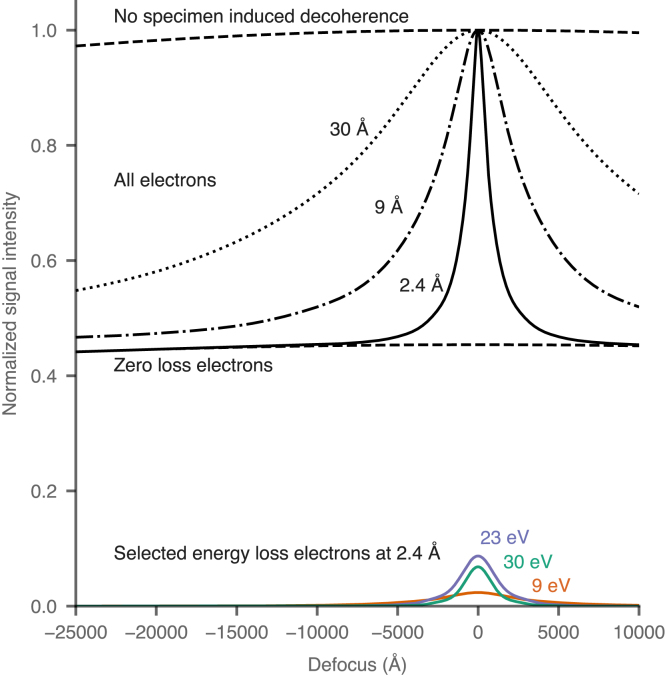
Simulations of the fading of power for different resolutions from a protein specimen embedded in amorphous water ice based on the measurements in Table 1. The specimen is 2000 Å thick and the protein to water ratio was taken as 30:70 with a simulated electron energy of 300 keV. Bottom curves show energy filtered phase contrast electrons at specific losses from Table 1. Middle dashed curve at a signal intensity of 0.44 shows the fading of all zero loss electrons (∼0-loss energy filtered imaging). Upper series of curves show the additional power at 2.4, 9 and 30 Å resolution from both elastic and inelastic phase contrast (∼ perfect $C_{c}$ correction and phase contrast). The FWHM is 1400, 5700, and 18800 Å for 2.4, 9 & 30 Å resolution respectively. The top dashed curve represents the total potential improvement in power if there were no specimen induced decoherence. Note: the sum of all energy losses and the zero loss electrons at 2.4 Å (including bottom colour plots & middle dashed curve) combine to produce the full 2.4 Å resolution curve (top solid line).

Specifically, simulations were used to predict the fading curves for other resolutions in a 2000 Å thick specimen of protein embedded in amorphous ice, where the ratio of water to protein was 70:30 (Fig. 5). Both $C_{c}$ and $C_{s}$ are set to zero in these simulations. These fading curves are also compared to zero loss energy filtered imaging (middle dashed line at a signal intensity of 0.44) and the hypothetical scenario in which there was no specimen induced decoherence (upper dashed line). It is clear that the fading with defocus is slower at lower resolutions, and thus more information can be recovered by including inelastic electrons at these frequencies. As the resolution increases, the drop-off becomes more severe, thus limiting the potential defocus range that could be used for high resolution imaging in the context of a $C_{c}$ corrected objective lens. Note the vast difference between the top dashed curve and the middle dashed curve at a signal intensity of 0.44. Real images collected with a $C_{c}$ corrected lens at a particular defocus will fall in between the two as indicated.

**Fig. 6:**
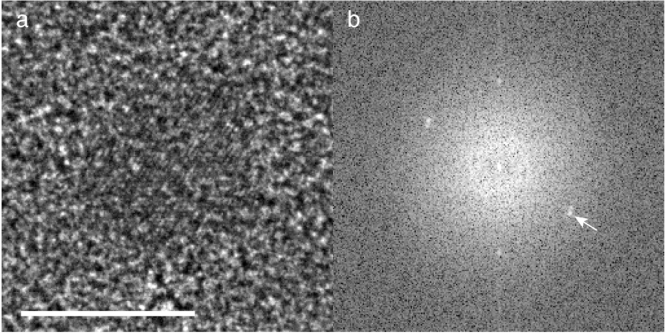
Representative micrograph (a) and FFT (b) of a gold particle on a 2000 Å thick carbon film, imaged in focus at 200 keV with $C_{c}$ correction. Fluence was ∼30 e${}^{-}/$Å${}^{2}$ in a 1 s exposure. Scale bar is 100 Å and arrow points to the 111 reflection at 2.4 Å.

An example in focus phase contrast micrograph of a 100 Å gold particle on a 2000 Å thick carbon film, taken on a $C_{c}$ corrected microscope, is shown in Fig. 6. Even at 200 keV, the atomic lattice is clearly resolved with marked contrast from the background. The level of contrast in the image of the particle is qualitatively consistent with the simulations, and imaging particles at high resolution in a thick specimen with a $C_{c}$ corrected microscope is clearly feasible. Further development is needed to quantify more accurately the contrast for cryogenically preserved specimens with $C_{c}$ correction and is beyond the scope of the current study.

## 5 Discussion

It is now of interest to consider the implications of specimen induced decoherence for imaging cryogenically preserved biological specimens and the potential improvement offered by chromatic aberration correction.

### 5.1 Imaging conditions imposed by the specimen

It is clear from the plots in Fig. 5 that to gain the maximum possible signal from incorporating inelastic electrons in a phase contrast image, it is necessary to remain as close to focus as possible. When imaging thick specimens, there will be high resolution elastic scattering from every layer of the specimen, but the additional signal from coherent inelastically scattered electrons will be limited to a narrower range close to focus as indicated by the falloff of the curves. Current practice in cryoET is to image with microns of defocus to enhance the low resolution contrast and then correct for the CTF during processing. This is still possible with $C_{c}$ correction but would entail a trade-off at high spatial frequencies as fewer inelastics will be added to the signal.

Alternatively, a means other than defocus could be used to generate phase contrast, such as a Zernike type phase plate to add a quarter wave shift to the transmitted wave. Several phase plates have been developed and successfully used for biological imaging [39], [40], [41], but all to date have had problems leading to substantial loss of signal, particularly at high resolutions [42].

Phase plate designs which overcome some of the previous problems by omitting any solid material susceptible to charging from the electron beam path, such as the laser phase plate [43], [44] and obstruction-free anamorphotic phase shifter [45], have the potential for greater success.

A fundamental issue to using a phase plate to generate contrast for inelastically scattered electrons is the specimen induced decoherence described here. All phase plates to date, including the laser phase plate design, rely on separating the forward scattered beam from the diffracted beams and inducing a phase shift between them. This entails that the phase plate will have a cut-on frequency, below which the phase contrast is no longer generated. This cut-on frequency would ordinarily be set as low as possible to maximise contrast at low spatial frequencies, which are important for identification and alignment of particles [46]. However, in the context of $C_{c}$ correction, an additional constraint is placed on the cut-on frequency because the forward scattered beam is broadened in the diffraction plane by inelastic scattering. This leads to an inevitable trade-off between the number of inelastic electrons incorporated in the signal and the minimum spatial frequency that is phase shifted. In spite of this trade-off, a phase plate would clearly be beneficial when used in conjunction with a $C_{c}$ corrector for imaging *in situ*.

Even with a $C_{c}$ corrector, the use of an energy filter to reduce noise must also be considered. If it were the case that all inelastic electrons remained coherent, there would be no need for energy filtering in the context of an achromatic lens [47] (equivalent to the upper dashed line in Fig. 5). But, as we have seen, the specimen induced decoherence severely limits the thickness range for which the electrons can contribute to phase contrast. This in turn implies that there is diminishing benefit in incorporating electrons as their energy loss increases since their angular distribution becomes progressively worse. This means it still may be beneficial to use an energy filter in conjunction with a $C_{c}$ corrector, albeit with a much wider slit width than is ordinarily used. The slit width will be dependent on a number of parameters, especially the specimen thickness and resolution of interest. It is not trivial to accurately model the noise reduction from energy filtering; future experiments with biological specimens at cryogenic temperatures are necessary to determine the optimum filter slit width for a variety of imaging conditions related to $C_{c}$ correction.

### 5.2 The range of thicknesses amenable to phase contrast

The benefits of using $C_{c}$ correction will be greatest for specimens between 1000 and 5000 Å thick. For specimens thinner than 1000 Å, the amount of inelastically scattered electrons generated will be small and thus are unlikely to significantly enhance the signal. There is some potential for $C_{c}$ correctors to be used to increase the information limit for very high resolution biological imaging (1 Å and below) by correctly focusing a wider range of energies from the electron source. However, as shown in Fig. S1, the signal enhancement even at 1 Å resolution will be modest and simply using existing high resolution objective lenses with limited tilt, which can have $C_{c}$s as low as 1 mm, could also be used to move the information limit below 1 Å resolution (compared to the $C_{c}=$ 2.7 mm lens used in the recent high resolution structures of apoferritin [48], [49]).

Specimens thicker than 5000 Å become increasingly difficult for phase contrast. It may still be possible to identify a structure within a narrow plane in a one micron thick specimen, but information about the regions above and below will be lost to decoherence. Beyond about one micron, multiple elastic scattering becomes dominant and will prevent any high resolution phase contrast. An alternative method for thick specimens is low-dose scanning transmission electron cryomicroscopy (cryo-STEM) with a broad probe [50], [51], [52], which offers an efficient and simple method for obtaining low resolution information.

### 5.3 Technical requirements for $C_{c}$ corrected imaging of biological specimens

Current chromatic aberration correctors are designed to work at electron energies up to 300 keV. In principle, higher energies are possible, but are more difficult to construct given the electrostatic elements present in the optical system. Using the information coefficient in Peet et al. 2019 [13], increasing the accelerating voltage from 300 kV to 500 kV for a 3000 Å thick specimen would yield an improvement of 4%. Increasing the electron energy also reduces the specimen induced decoherence by a similar amount (4% for 300 keV to 500 keV on a 3000 Å thick specimen). Thus the potential improvements in going to higher beam energies than 300 keV are relatively modest in comparison to the technical challenges posed by creating correctors and phase plates at these higher voltages. It is also worth noting that the current $C_{c}$/$C_{s}$ correctors were developed with very high resolution (∼0.5 Å) imaging of atomically thin specimens in mind [53]. But in the context of biological imaging *in situ*, resolutions beyond 2 Å are unlikely to contribute to identification and alignment, offering an opportunity to reduce the complexity and cost of a corrector designed for biological applications.

### 5.4 Minimum molecular mass identifiable in situ

The largest potential benefit from $C_{c}$ correction in biology is the identification of small proteins *in situ*. A question we can now ask is: what is the minimum molecular mass of a protein that can be detected in a given thickness of specimen? In the absence of scattering from amorphous ice, a value of 38 kDa was predicted by Henderson in 1995 [54]. Using a similar calculation with more recently measured differential cross sections, we have reached a similar value of 42 kDa using the same assumptions. This is a soft limit that could be improved with more data, as outlined in Appendix B. With this analysis as a starting point, we now predict the loss of signal as the thickness increases, in the following way:

We first define the minimum molecular mass identifiable in a vitrified biological specimen of thickness t as

(5) $$M_{min}=\frac{M_{0}+M_{blur}}{e^{-ta\left(\lambda_{i}^{-1}+\lambda_{e}^{-1}\right)}}$$

where $\lambda_{i}$ is inelastic mean free path length in the specimen, $\lambda_{e}$ is the elastic mean free path length, $M_{0}$ is the theoretical minimum molecular mass at zero thickness, $M_{blur}$ is a term that accounts for losses of signal like imperfect detector efficiency and other forms of noise not in the model such as movement of the specimen during imaging. It will be close to zero in the case of thin, near-ideal single particle specimens but may be of order 100 kDa for thick specimens previously milled with ion beams and imaged at tilt. The constant a is a correction factor related to the size of the protein and is equal to 1.05 (see Appendix B).

**Fig. 7:**
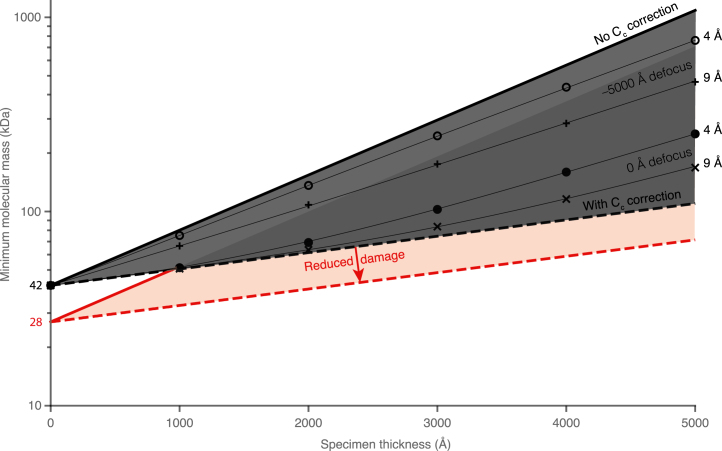
Plots of the minimum protein molecular mass identifiable *in situ*. The solid lines represent phase contrast imaging without $C_{c}$ correction (Eq. (5)) and the dashed lines with $C_{c}$ correction (Eq. (6) and $E\left(\vec{k}\right)$ equal to 1). The data points are from Eq. (6) integrated for the range of defocus values across the specimen and centred on either zero defocus (solid circles and ×’s) or at −5000 Å defocus (open circles and ＋’s). Thin black lines guide the eye and show a range of resolutions which might be required for unique identification and alignment of a specific molecule or complex under each defocus. The zero defocus condition depends upon phase contrast generated by some means other than defocus, e.g. a phase plate without loss. In the limit of close to zero thickness, the single particle conditions are recovered and correspond to the limit in Henderson (1995) [54]. The red dashed line and shaded region indicate the improvement that could be expected by reducing the rate of radiation damage by a factor of 1.5, as may be possible with additional cooling of the specimen to temperatures closer to 0K than are currently used [55].

For a $C_{c}$ corrected microscope, Eq. (5) can be modified to include inelastic scattering according to

(6) $$M_{min}=\frac{M_{0}+M_{blur}}{E\left(\vec{k}\right)e^{-ta\lambda_{e}^{-1}}+\left(1-E\left(\vec{k}\right)\right)e^{-ta\left(\lambda_{i}^{-1}+\lambda_{e}^{-1}\right)}}$$

where $E\left(\vec{k}\right)$ is the fractional signal from inelastically scattered electrons at spatial frequency k as a result of the spatial coherence envelope function (Eq. (4)). The above equations were plotted (with $M_{blur}=0$) for thicknesses up to 5000 Å and are shown in Fig. 7. It is clear that the particular advantage of $C_{c}$ correction will be in increasing the thickness range over which sub-500 kDa proteins can be identified.

Identification of proteins in thick biological specimens can be performed using both 3D template matching [56] and 2D template matching [57]. By using 2D template matching, the signal lost through increasing specimen thickness by tilting is reduced and thus the high resolution signal is maximised. In addition, a tilted specimen will be at an angle relative to the plane of focus, which will severely restrict the lateral range for which enhancement in signal from $C_{c}$ correction in a tomographic reconstruction is possible. The theoretical minimum molecular mass predicted here is significantly lower than the 150 kDa estimate from Rickgaur et al. (2017) [12], but consistent with current ability to successfully align single particle images with each other in thin specimens, which is essentially the same process as 2D template matching. This indicates that there are improvements to the minimum molecular mass to be had which will bring 2D template matching in line with the minimum molecular mass that is currently feasible in single particle structure determination. In particular, maximum likelihood-based methods of aligning specific particles within a heterogenous specimen, which have been successful in single particle reconstruction techniques, may be useful here as well. One significant unknown is the range of spatial frequencies required for successful identification of a known structure in a micrograph; hence the ranges plotted in Fig. 7. These will be dependent on the form factors of the molecules to be identified and can likely be measured to some extent empirically on a range of model specimens. It is also of note that since the specimen induced decoherence severely limits the defocus range for which the signal is enhanced, a Zernike-type phase plate would be particularly useful for $C_{c}$ corrected imaging of thick specimens.

Reductions in $M_{blur}$ are likely to have contributions from reduced specimen movement [32], [58], [59], and improvements in alignment algorithms and image simulations from atomic structures. The radiation damage could also be reduced using liquid helium cooling [55], which lowers $M_{0}$ as indicated in Fig. 7. Taken together, the incorporation of inelastically scattered electrons in conjunction with other technological developments already in progress, will potentially allow the identification and localisation of sub-100 kDa proteins *in situ*.

## Declaration of Competing Interest

The authors declare that they have no known competing financial interests or personal relationships that could have appeared to influence the work reported in this paper.

## Appendix A Derivation of spatial coherence envelope function

The phase coherence function Γ between any two points on the source was defined as

(A.1) $$\Gamma \left(\vec{r_{1}}-\vec{r_{2}}\right)=\int s\left(\vec{q}\right)exp\left(-2i\pi \vec{q}\left(\vec{r_{1}}-\vec{r_{2}}\right)\right)d\vec{q}$$

where s(q) is the intensity distribution in the source plane as a function of spatial frequency q. The wave aberration function was defined as

(A.2) $$\chi \left(\vec{k}+\vec{q}\right)\approx \chi \left(\vec{k}\right)+\vec{q}\nabla \chi \left(\vec{k}\right)+\cdots$$

The higher order terms were shown by Frank to be insignificant [19]. He defines the envelope function as

(A.3) $$E\left(\vec{k}\right)=\Gamma \left(\nabla \chi \left(\vec{k}\right)\right)$$

We can now use the 2D equation for a Lorentzian in polar coordinates and substitute into Eq. (A.1) to get

$$E\left(\vec{k}\right)=\frac{1}{2\pi }\int_{0}^{2\pi }\int_{0}^{\infty }\frac{\gamma }{{\left({\left(q\sin\psi \right)}^{2}+{\left(q\cos\psi \right)}^{2}+\gamma^{2}\right)}^{\frac{3}{2}}}exp\left(-2\pi iq\left|\nabla \chi \left(k\right)\right|\left(\psi -\psi^{\prime }\right)\right)q\;dqd\psi$$

where γ is the half width at half maximum of the Lorentzian distribution. On integration we have

(A.4) $$E\left(\vec{k}\right)=\int_{0}^{\infty }\frac{\gamma }{{\left(q^{2}+\gamma^{2}\right)}^{\frac{3}{2}}}J_{0}\left(2\pi q\left|\nabla \chi \left(k\right)\right|\right)q\;dq$$

where $J_{0}$ is the 0th order Bessel function. The limit can be set as the cutoff for plasmon scattering, which is estimated to be $\sqrt{2\gamma }=q_{c}$ [37]. Substituting in also that $\left|\nabla \chi \left(k\right)\right|=\Delta z\theta +C_{s}\theta^{3}$, we find

(A.5) $$E\left(\vec{k}\right)=\int_{0}^{q_{c}}\frac{\gamma }{{\left(q^{2}+\gamma^{2}\right)}^{\frac{3}{2}}}J_{0}\left(2\pi q\left(\Delta z\theta +C_{s}\theta^{3}\right)\right)q\;dq$$

## Appendix B Determination of minimum molecular mass

We apply a similar analysis to that in Henderson 1995 [54] to determine the theoretical minimum detectable molecular mass as a function of thickness. For a given protein molecular mass (M), the protein density is calculated according to the empirical equation presented in Fischer et al. 2009 [60]:

(B.1) $$\rho =1.410+0.145e^{-\frac{M}{13}}$$

where ρ is the density in g/cm^3^, and M is the molecular mass in kDa. The protein diameter, D, in Ångstroms is then

(B.2) $$D=2{\left(\frac{3M}{4\pi \rho N_{A}\times 10^{-27}}\right)}^{\frac{1}{3}}$$

where $N_{A}$ is Avogrado’s number.

The differential elastic scattering cross sections from ELSEPA [61] were used to determine the fraction of electrons elastically scattered to a particular resolution for an incident energy of 300 keV. In this case, 3 Å was used. The number of amino acids in the protein was calculated as M/0.11, which assumed an average amino acid molecular mass of 110 Da. ELSEPA was also used to calculate the total elastic scattering cross section of a protein, assuming an average amino acid composition of 4.9 C, 1.38 N, 1.49 O, and 7.7 H. The fraction of elastically scattered to a particular resolution, f, is then

(B.3) $$f=\beta \left(\frac{\sigma_{e}}{D^{2}}\right)$$

where $\sigma_{e}$ is the total elastic scattering cross section of the protein, and β is the fraction of electrons that elastically scatter within the selected resolution, d. The total number of electrons incident on the specimen is then

(B.4) $$N=\pi {\left(\frac{D}{2}\right)}^{2}N_{e}$$

where $N_{e}$ is the exposure in e${}^{-}/$Å ^2^ at the dose limit. Since each Fourier component is composed of two Friedel related spots, the intensity is calculated by multiplying the electron fraction by two times the number of incident electrons

(B.5) I=2Nf

The major contribution to noise in low-dose imaging is shot noise [62]. This scales as the square root of the intensity, so the signal to noise ratio (SNR) scales in proportion to $\sqrt{I}$.

The value to use for $N_{e}$ is difficult to estimate. The loss of signal with increasing exposure is dependent on the motion of the particles, and more strongly, on radiation damage. The rates of some of these phenomena are well known, and could thus be included in the calculations. The amount of pseudo-Brownian motion of water was experimentally determined as 1 Å per e${}^{-}/$Å ^2^ [63]. The diffusion coefficient reduces linearly as particle diameter increases, which is about 2.7 Å for a water molecule and calculated in Eq. (B.2) for proteins. The mean squared displacement (MSD) will scale linearly with the diffusion coefficient. The MSD, $\langle u^{2}\rangle$, can then be converted to a B-factor according to

(B.6) $$B_{motion}=\frac{8\pi^{2}}{3}\langle u^{2}\rangle$$

The derivative of the B-factor with respect to fluence in e${}^{-}/$Å ^2^ is then

(B.7) $${\overset{˙}{B}}_{motion}=\frac{7.2\pi^{2}}{D}$$

For radiation damage, recent experiments indicate a B-factor (${\overset{˙}{B}}_{rad}$) of approximately 5 Å${}^{2}$ per e${}^{-}/$Å ^2^ is appropriate [59]. The reduction in intensity due to radiation damage at resolution d for a fluence of $N_{e}$ is thus

(B.8) $$\propto \exp\left(-\frac{N_{e}\left({\overset{˙}{B}}_{rad}+{\overset{˙}{B}}_{motion}\right)}{2d^{2}}\right)$$

However, since the reduction in intensity is strongly resolution dependent, it would require knowledge of the extent to which each resolution contributes to protein identification to be able to apply these B-factors. More experimental work will be needed to fully understand this, so it will not be considered further here. We will use a value of 5 e${}^{-}/$Å ^2^ for $N_{e}$, as was used in Henderson 1995. Future experiments are planned to better understand this relationship, as well as the role of various frequency bands in particle alignment in this context. Once the resolution dependence has been determined, as well as applying these B-factors, we can also use the number of electrons scattered to each particular resolution (calculated by ELSEPA) and apply the relevant contrast transfer functions.

Again following Henderson 1995, we determine if this SNR is high enough to detect a particle by comparing it to that which would be expected by chance. The number of cross-correlations that would need to be examined to determine the position of a particle to within ± 0.1 pixels is approximately

(B.9) $${\left(\frac{10D}{d}\right)}^{2}$$

For a thick specimen, it may be necessary to also search for the position in z to get an accurate enough defocus. In this case, the equation will instead scale to the third power. To determine the orientation, the number of cross correlation coefficients to be examined is

(B.10) $${\left(\frac{10\pi D}{d}\right)}^{3}$$

The total number of cross correlation coefficients to examine for a thin specimen is then

(B.11) $$\pi^{3}\times 10^{5}{\left(\frac{D}{d}\right)}^{5}$$

Assuming that noise is random and follows a normal distribution, the SNR must be larger than x, where

(B.12) $$erfc\left(x/\sqrt{2}\right)=\frac{1}{\pi^{3}\times 10^{5}{\left(\frac{D}{d}\right)}^{5}}$$

(B.13) $$erfc\left(t\right)=\frac{1}{\sqrt{\pi }\int_{t}^{\infty }e^{-z^{2}}}$$

thus

(B.14) $$x=\sqrt{2}erfc^{-1}\left[{\left(\frac{D}{d}\right)}^{5}/\left(\pi^{3}\times 10^{5}\right)\right]$$

If we also have to search in z, this becomes

(B.15) $$x=\sqrt{2}erfc^{-1}\left[{\left(\frac{D}{d}\right)}^{6}/\left(\pi^{3}\times 10^{6}\right)\right]$$

The minimum detectable molecular mass is then the smallest mass for which the SNR is greater than x. Using this method, the minimum molecular mass detectable is determined as 42 kDa. This would increase to 50 kDa if three dimensions are considered. We call this value $M_{0}$.

We can then account for the losses in *signal* as a result of an increase in thickness. These losses are a result of both elastic and inelastic scattering in the surrounding material. Specifically, the loss of electrons in the forward scattered beam can be described by adding terms to Eq. (B.3). This takes the form of a Poisson distribution according to

(B.16) $$e^{-t\left(\lambda_{i}^{-1}+\lambda_{e}^{-1}\right)}$$

where t is the specimen thickness, $\lambda_{i}$ is the inelastic mean free path length and $\lambda_{e}$ is the elastic mean free path length. The minimum molecular mass, $M_{min}$, can then approximated using the following simple equation

(B.17) $$M_{min}=\frac{M_{0}+M_{blur}}{e^{-ta\left(\lambda_{i}^{-1}+\lambda_{e}^{-1}\right)}}$$

where $M_{0}$ is the theoretical minimum molecular mass at zero thickness, and $M_{blur}$ accounts for losses of signal not included in the model (see also Section 5.4). The correction factor a is determined in the following way:

1. The value of x is determined for a range of different particle diameters (and thus masses) using Eq. (B.14) and the density of protein (from Eqs. (B.1) & (B.2)).
2. The SNR is determined for these same masses in a range of thicknesses (0–5000 Å) using Eq. (B.3) to (B.5).
3. For each thickness, the minimum molecular mass ($M_{min}$) in which SNR > x is then taken as a point in the plot of mass vs. thickness (solid lines in Fig. 7).
4. A fit is then performed to determine the value of a according to the following

(B.18) $$\ln\left(M_{min}\right)=\ln\left(M_{0}+M_{blur}\right)+at\left(\lambda_{i}^{-1}+\lambda_{e}^{-1}\right)$$

with $M_{blur}$ set to zero. The factor a, which is equal to 1.05, allows a simple expression for the $M_{min}$ and physically arises from the increase in x vs. molecular mass from Eq. (B.14) as the protein size of interest increases. It was the same for both the $C_{c}$ corrected and non-$C_{c}$ corrected cases, as well as being the same for considering two or three dimensions in the determination of x (Eqs. (B.14), (B.15) respectively).

For a $C_{c}$ corrected microscope, the electrons that have undergone multiple elastic scattering are still lost, but the inelastics can be partially recovered, depending on the spatial coherence. Therefore, the fraction of electrons that contribute to signal becomes

(B.19) $$E\left(\vec{k}\right)e^{-t\lambda_{e}^{-1}}+\left(1-E\left(\vec{k}\right)\right)e^{-t\left(\lambda_{i}^{-1}+\lambda_{e}^{-1}\right)}$$

where $E\left(\vec{k}\right)$ is the fractional signal remaining from inelastically scattered electrons at spatial frequency k as a result of the spatial coherence envelope function (Eq. (4)). We thus obtain an expression for $M_{min}$ assuming perfect $C_{c}$ correction

(B.20) $$M_{min}=\frac{M_{0}+M_{blur}}{E\left(\vec{k}\right)e^{-ta\lambda_{e}^{-1}}+\left(1-E\left(\vec{k}\right)\right)e^{-ta\left(\lambda_{i}^{-1}+\lambda_{e}^{-1}\right)}}$$

which is the same as Eq. (6) in the main text.

## Appendix C Supplementary data

Fig. S1 Figure showing the temporal coherence envelope function with different objective lenses and source energy spreads.
