# Supplementary material for: Imaging biological macromolecules in thick specimens: The role of inelastic scattering in cryoEM
Source: Ultramicroscopy. 2022 Jul;237:None. doi: 10.1016/j.ultramic.2022.113510 (PMC9355893; doi:10.1016/j.ultramic.2022.113510)
Supplement: Fig. S1 — Figure showing the temporal coherence envelope function with different objective lenses and source energy spreads. [file mmc1.pdf]

Supplementary Figure

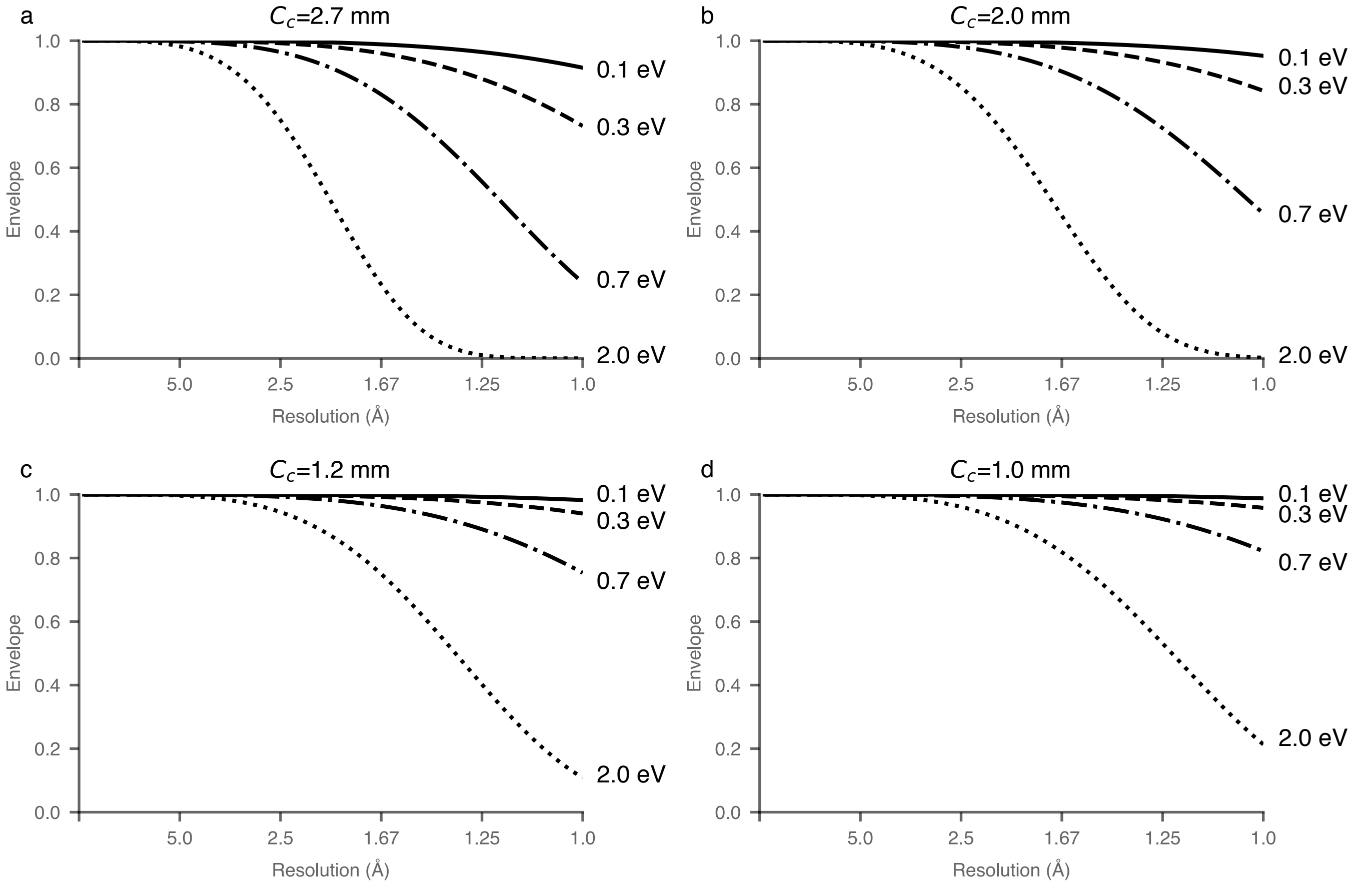

Fig. S1: Plots of the temporal coherence function for a range of objective lens specifications and source energy spreads at 300 keV. (a) Corresponds to the parameters of a FEI/TFS C-TWIN which is commonly found in Titan Krios microscopes configured for cryomicroscopy. (b) to a TWIN, (c) to a S-TWIN, and (d) to a U-TWIN. The C_c of these lenses is 2.7, 2.0, 1.2 and 1.0 mm respectively [1, 64]. 0.1 eV is taken as the energy spread of a monochromated beam [65], 0.3 eV of a cold FEG [66], and 0.8 eV of a Schottky FEG [1].
